# Correlation analysis of the vertebral compression degree and CT HU value in elderly patients with osteoporotic thoracolumbar fractures

**DOI:** 10.1186/s13018-023-03941-z

**Published:** 2023-06-26

**Authors:** Cheng Li, Xing-ming Lai, Nian Liu, Yang Lin, Wei Hu

**Affiliations:** Department of Orthopaedics, Fifth Clinical Medical College, Guilin Medical University, No. 12 Wenming Road, Guilin, 541002 Guangxi Province China

**Keywords:** CT hounsfield unit, Osteoporotic thoracolumbar fractures, BMD, Cancellous bone

## Abstract

**Background:**

To explore the correlation of the vertebral compression degree and cancellous bone CT HU in elderly patients with osteoporotic thoracolumbar fractures.

**Methods:**

Elderly patients with single-segment vertebral fragility fractures were retrospectively reviewed. All patients experienced a low-energy trauma and underwent thoracolumbar MRI. The consistency of measurement between two spine surgeons was evaluated. The average CT HU value of the adjacent vertebral body was used instead.

**Results:**

A total of 54 patients were included in the final analysis. The patients’ average age was 70.39 ± 8.53 years, and the average CT HU value was 72.78 ± 29.75 HU. The average vertebral compression ratio was 0.57 ± 0.16. Measurements showed both good intrarater repeatability and good interrater reproducibility of the vertebral compression ratio (ICC = 0.978). The degree of vertebral compression in thoracolumbar osteoporotic fractures was strongly positively correlated with the cancellous bone CT HU value (*P* < 0.01).

**Conclusions:**

The local bone quality as evaluated by the CT HU value is an important factor affecting the degree of compression in osteoporotic vertebral fractures. This study provides quantitative evidence that a greater compression ratio with thoracolumbar osteoporotic fractures was associated with lower bone density in elderly patients. Further longitudinal studies with larger cohorts are needed to verify this relationship.

## Background

With ageing of the population, osteoporosis is becoming a global problem and currently affects approximately 200 million people worldwide [1]. Vertebral fractures are the most common osteoporotic fractures, followed by fractures of the proximal femur and distal radius. Due to specific spinal biomechanics [2], the thoracolumbar vertebral bodies (T10–L2) are the most easily fractured vertebral bodies in spinal trauma.

Dual-energy X-ray absorptiometry (DXA) is currently considered to be the gold standard for bone mineral density (BMD) quantification and has been shown to correlate with fracture risk and therapeutic efficacy [3]. However, although BMD is a risk factor for fracture, some fragility fractures occur in individuals with BMD T values above the − 2.5 threshold, which suggests that BMD has limited clinical value in predicting osteoporosis [4, 5]. BMD determination is also the most commonly used method for evaluating bone density in spinal surgery. Elderly patients with osteoporotic fractures tend to have concomitant degenerative disease; however, lumbar degenerative diseases may lead to artificially increased DXA measurements with missing diagnosis of osteoporosis. It has been proven [6] that the prevalence of osteoporosis is high in patients with lumbar degenerative disease, as is the rate of missed diagnosis by DXA. Thus, DXA alone is insufficient for assessing BMD in patients with lumbar degenerative disease.

Genant et al. [7] proposed a semiquantitative method for classifying osteoporotic vertebral compression fractures, which is now commonly used. This approach involves the use of lateral thoracolumbar X-rays to determine the degree of vertebral compression. However, the method is sensitive to the angle of exposure, patient position, morphology of the vertebral body, and experience of the X-ray technician, which may cause small errors in determining the degree of vertebral body compression in some cases. In contrast, CT allows sagittal alignment evaluation, and the resulting images are sufficient to determine the degree of compression. Because of the limitations of the semiquantitative approach in assessing vertebral compression, we applied three-dimensional CT to assess vertebral compression on the sagittal plane in cases of fragility fractures.

Studies [8, 9] have found that the CT Hounsfield unit (HU) value of the vertebral trabecular (noncortical) region can reflect the BMD and that routine CT examination can be used to identify patients with osteoporosis. Thus, it is recommended that the CT HU value of the vertebral body be considered along with the BMD score to improve the accuracy of the BMD assessment. CT HU value can also be used to assess the risk of thoracolumbar vertebral fragility fractures. With lower vertebral body CT HU value, the risk of vertebral body fragility fracture increases, as does the likelihood of multiple vertebral fractures [10]. However, there has been no research on using vertebral CT HU value to quantitatively analyze the severity of vertebral body osteoporotic fractures. For this reason, this study aimed to further explore the relationship between the vertebral cancellous bone CT HU value and osteoporotic vertebral compression fracture severity.

## Methods

### Patient cohort

This study was reviewed and approved by the Fifth Clinical Medical College of Guilin Medical University. The requirement for informed consent was waived because this study was retrospective. All methods were performed following relevant guidelines and regulations.

All patient data were retrieved retrospectively from our institute’s radiology information system. We reviewed the files of elderly patients treated for acute vertebral fragility fractures at our department from January 2019 to December 2020. All included cases were followed up for at least one year. The inclusion criteria were as follows: (1) acute vertebral compression fracture affecting a single segment from T10 to L2; (2) injury due to low-energy trauma, with no obvious history of high-energy trauma; (3) clear MRI and CT images (which had not motion artifacts), and diagnosis of osteoporosis or osteopenia by DXA; (4) no history of spinal surgery associated with disease. The exclusion criteria were as follows: (1) single-segment vertebral compression fracture outside the thoracolumbar spine or multisegmental vertebral compression fractures; (2) clear history of high-energy trauma; (3) no concurrent MRI, CT, or DXA scan; (4) old vertebral compression fracture; (5) fracture due to secondary osteoporosis (oral hormone usage), or pathology (metastases); (6) postoperative status, or known history of disability; (7) Kummel disease, stiff spine or diffuse idiopathic skeletal hyperostosis (DISH) morphology; and (8) history of spinal surgery associated with disease.

### CT and measurements

**Fig. 1:**
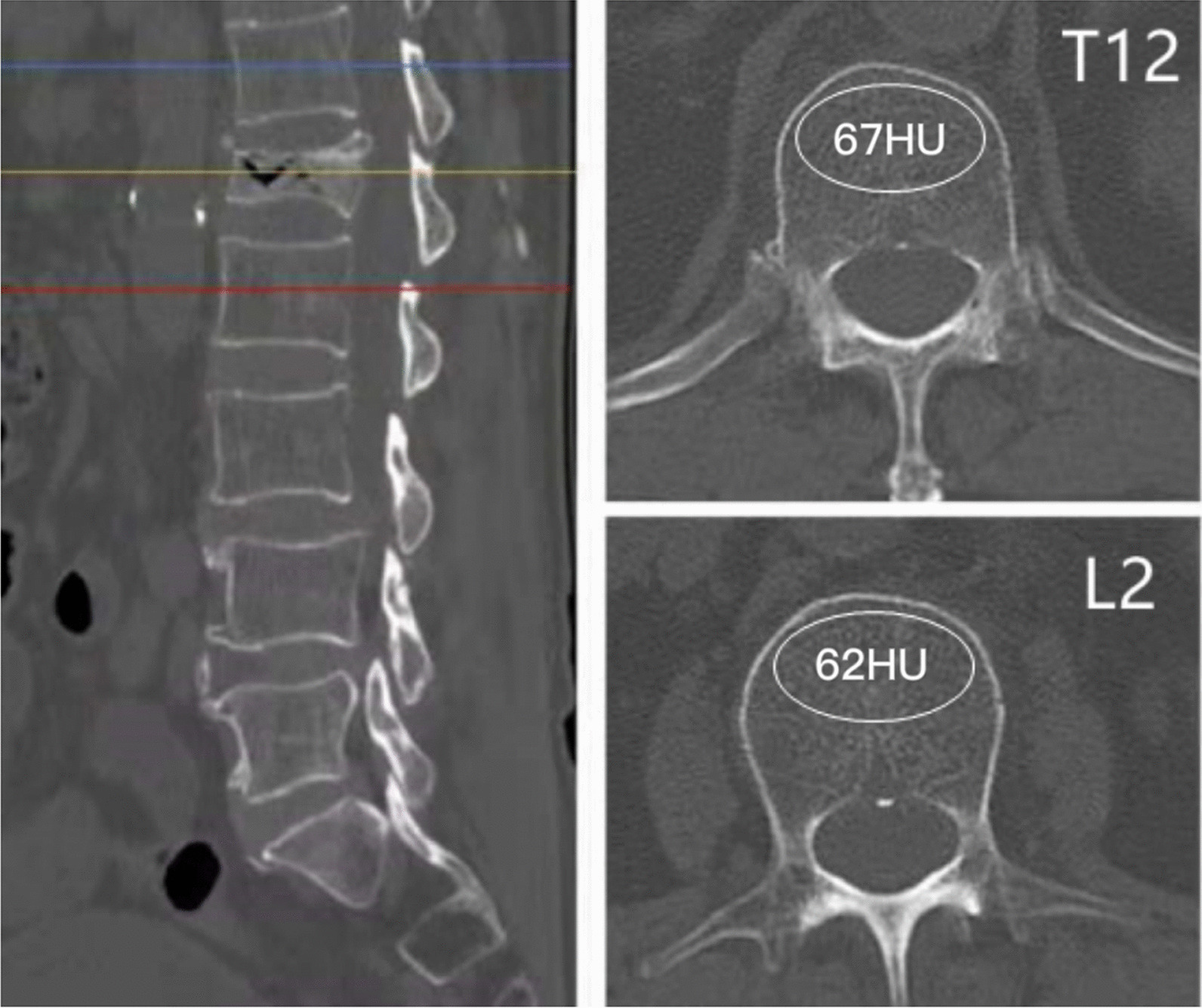
An 81-year-old male with a compression fracture of the L1 vertebral body. The average cancellous bone CT HU value of the T12 and L2 vertebral bodies was determined because of severe compression

**Fig. 2:**
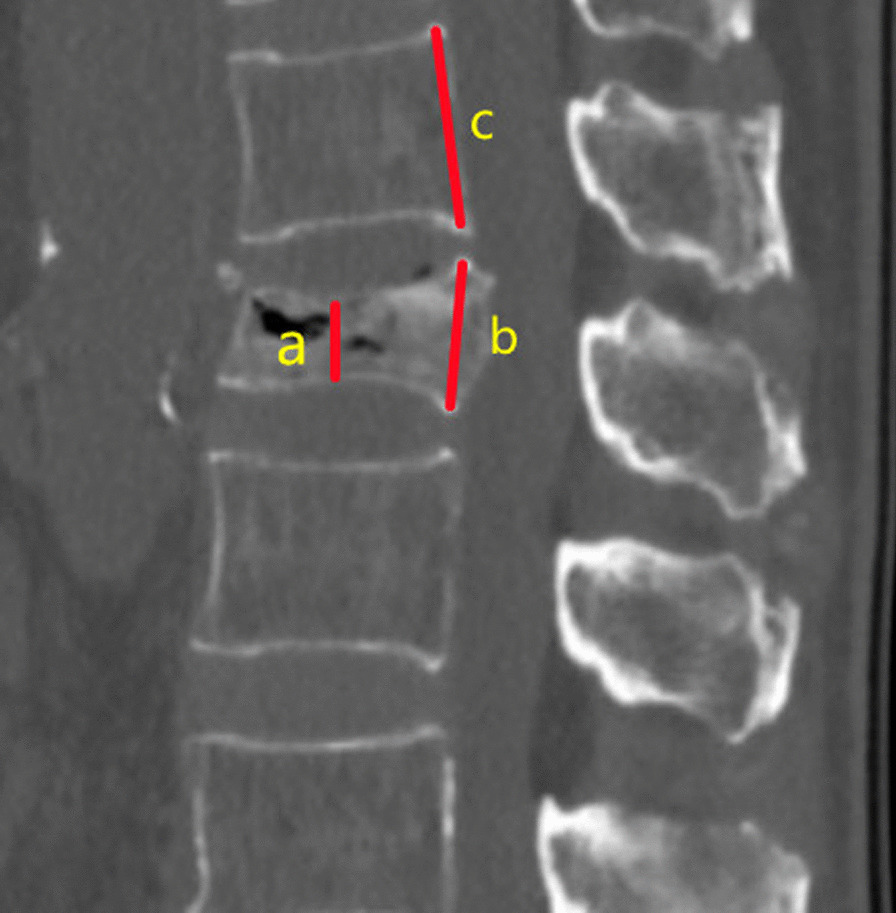
In the case of whole-body compression, the degree of vertebral compression was determined by a/c rather than a/b

All MRI scans were performed using either of two 1.5-T scanners (Signa, GE Healthcare). The thoracolumbar vertebral body was chosen as the site for CT HU value measurement based on the preoperative three-dimensional spinal CT reconstruction (American GE Company, Light Speed VCT, scanning conditions: tube voltage: 120 kV, tube current: 355 mA, slice thickness: 5 mm, slice spacing: 5 mm, bone window width: 2000 HU, window level: 350 HU). The picture archiving and communication system (PACS) was used to measure the vertebral compression degree and cancellous bone CT HU value. An oval region of interest (ROI) was placed on the middle axial image within the trabecular bone of the thoracolumbar vertebral body, avoiding the vertebral venous plexus, cortical bone, and any areas of hyperosteogeny or artefacts [11–13]. Two spinal surgeons independently measured the CT HU value of the cancellous bone of the fractured vertebra on a single axial CT image at the appropriate level by manually placing the ROI, and the analysis showed excellent interobserver and intraobserver reliability for the measurements (Interobserver reliability ICCs ranged from 0.70 to 0.91, intraobserver reliability ICCs over 0.8 (*P* < 0.001) [14, 15]. If the vertebral body showed a compression fracture on the sagittal plane of the three-dimensional CT reconstruction, the average CT HU value of the adjacent vertebral body was used instead (Fig. 1). Commonly, osteoporotic vertebral compression fractures compression ratio is the height of the most compressed part of vertebral body to that of the posterior edge of the vertebral body. And if whole-body compression occurred, osteoporotic vertebral compression fractures compression ratio is the height of the most compressed part of vertebral baby to that of the posterior edge of the superior vertebral body (Fig. 2) [7, 10].

### Statistical analyses

The statistical analysis in this study was conducted using SPSS version 26 (SPSS, USA). Measurement data are shown as the means ± standard deviations. Two spinal surgeons measured data for consistency analysis, which was performed by determining the intraclass correlation coefficient (*ICC*). The Spearman correlation test was applied for correlation analysis of the vertebral compression degree and cancellous bone CT HU value in thoracolumbar osteoporotic fractures. *P* < 0.01 was considered to indicate a statistically significant difference.

## Results

A total of 54 elderly patients, including 39 females and 15 males, were included in the final analysis. Based on the AO Spine OF Classification [16], there were 38 type A1, 8 type A2 and 8 type A3 vertebral body fractures, including 24 L1, 17 T12, 8 L2, 2 T11, and 3 T10 osteoporotic vertebral fractures. The patients’ average age was 70.39 ± 8.53 years (53–90 years), and the average CT HU value was 72.78 ± 29.75 HU (15.50–142.50 HU). The average vertebral compression ratio was 0.57 ± 0.16 (0.15–0.92) (Table 1).

**Table 1:** Analysis of general data of 54 patients with thoracolumbar fragility fracture

Indicator	All (54)
Age	70.39 ± 8.53
Gender	
Female	39
Male	15
Average CT HU value	72.78 ± 29.75
Fractured vertebral body	
T10	3
T11	2
T12	17
L1	24
L2	8
AO spine of classification	
A1	38
A2	8
A3	8
Genant classification	
Grade 0	4
Grade 1	4
Grade 2	16
Grade 3	30

### Consistency test

The consistency of the measurements obtained by the two spinal surgeons was tested, as was the consistency of the thoracolumbar fracture compression ratios (ICC = 0.978 > 0.75). The consistency of the data measured by the two spinal surgeons was high (Table 2).

**Table 2:** Consistency test of fracture vertebral compression ratio measured by two spinal surgeons

	Intra-class correlation	95% confidence interval		*F*-test with true value 0			
		Lower limit	Upper limit	Value	*df*1	*df*2	Significance
Single measurement	0.957	0.927	0.975	45.460	53	53	0.000
Average measurement	0.978	0.966	0.987	45.460	53	53	0.000

### Correlation between vertebral compression degree and cancellous bone CT HU value in thoracolumbar osteoporotic fractures

**Fig. 3:**
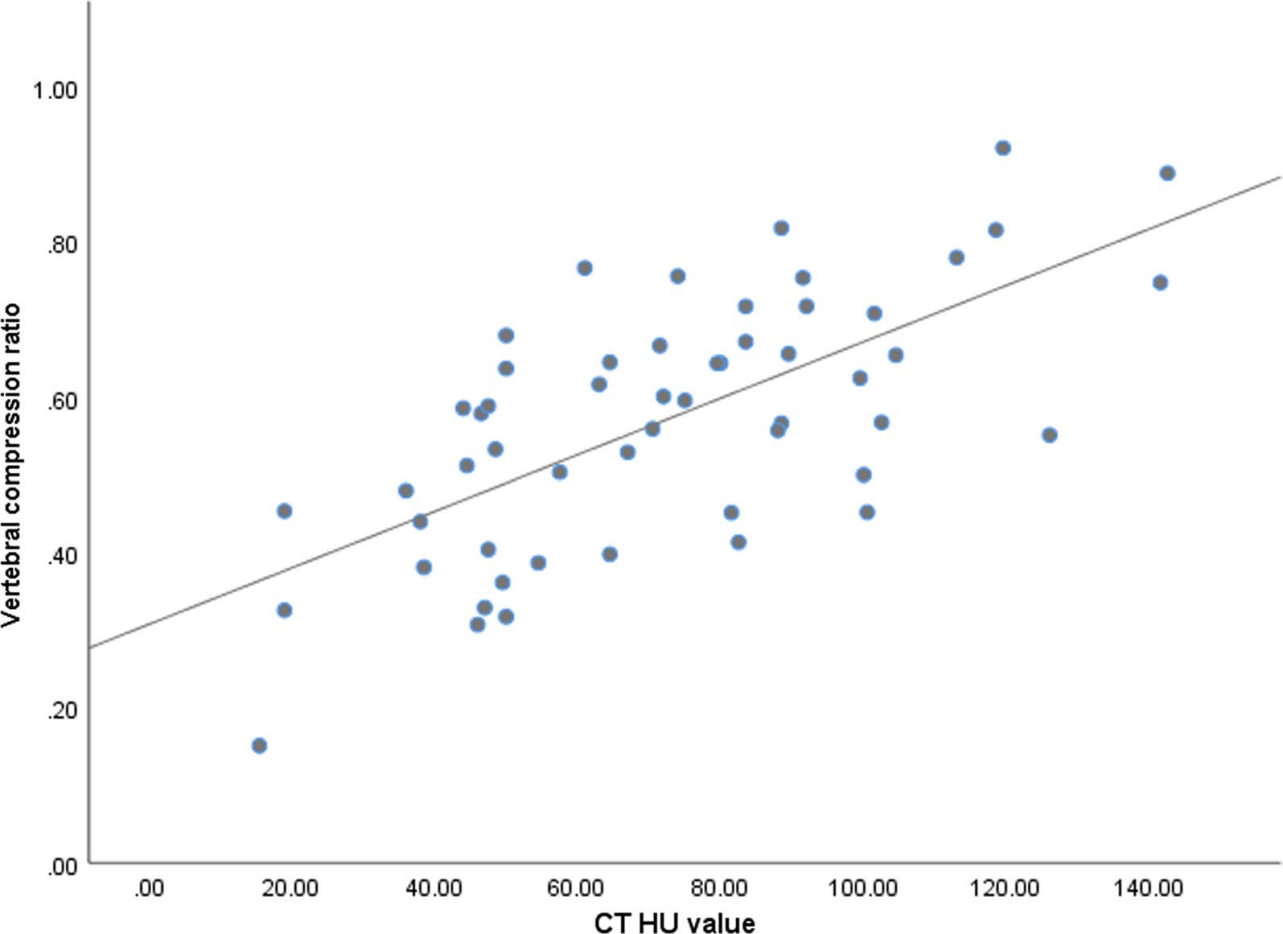
Strong positive correlation of the degree of vertebral compression with the CT HU value of cancellous bone in thoracolumbar osteoporotic fractures (*R*^2^ = 0.454)

The degree of vertebral compression in thoracolumbar osteoporotic fractures was strongly positively correlated with the CT HU value of cancellous bone (*P* < 0.01), with a correlation coefficient of *r* = 0.628 (Table 3, Fig. 3). A greater compression ratio was associated with lower bone density.

**Table 3:** Spearman correlation of vertebral compression ratio and CT HU value

	Vertebral compression ratio	CT HU value
Vertebral compression ratio		
Spearman correlation	1.000	0.628**
P		0.000
N	54	54
CT HU value		
Spearman correlation	0.628**	1.000
P	0.000	
N	54	54

**At the 0.01 level (two-tailed), the correlation is significant

## Discussion

Our study demonstrates that in low-energy osteoporotic fractures of thoracolumbar vertebrae (T10-L2), with decreases in the cancellous bone CT HU value, the degree of compression becomes increasingly obvious. There was a typical linear relationship between the degree of vertebral compression and the CT HU value of cancellous bone. In our study, all patients had experienced a low-energy trauma and were diagnosed with osteoporosis or osteopenia based on DXA for BMD assessment after admission. MRI is currently the preferred imaging method for the diagnosis and treatment of fragility vertebral fractures [17], and we used thoracolumbar MRI to assess abnormal signal indicating oedema and whether multiple vertebral bodies were fractured.

The CT HU value represents the attenuation of X-rays after they pass through and are absorbed by tissue. The HU, i.e., the Hounsfield unit, is used for measuring the density of a local tissue or organ in the human body. The CT HU value is based on the properties of water and is equal to the difference between the attenuation coefficient of the substance and the absorption coefficient of water multiplied by 1000 after determining the ratio to the attenuation coefficient of water [13]. The CT HU value reflects the density of a material; the higher the density of a material is, the higher the CT HU value, and the human skeleton is no exception. The HU value of bone typically ranges from 300 to 3000 [18]. Compared with DXA examination, CT HU value measurement for evaluating BMD confers several obvious advantages. (1) DXA cannot distinguish between cortical and cancellous bone [9]. Moreover, a previous study [19] has suggested that cancellous bone plays a more important role than cortical bone in the process of spinal loadbearing and compression fractures. (2) Conventional DXA can only be used to determine the BMD of the lumbar spine and hip, which are easily fractured sites. However, CT is not limited by the examination site, and there are many patients at a high risk of vertebral fragility fractures who have not undergone standardized a BMD assessment. Additionally, many elderly people have undergone clinical CT examinations for various diseases (chest CT, abdominal and pelvic CT, spinal CT, urinary CT, etc.), and these existing CT images could be used to measure the vertebral body CT HU value, assess the BMD, and easily and quickly identify patients at a high risk of osteoporosis and fragility vertebral fractures. (3) The prevalence of multidetector-row helical CT machines in medical institutions is significantly higher than that of DXA systems; at the same time, measurement of the HU value is convenient and shows good to excellent reliability between observers, all of which makes this method simple and easy to popularize [14].

As such, this study suggests that the CT HU value is more intuitive than the DXA T value as an indicator of the vertebral body bone mass. The patients included in this study all had osteoporosis; the CT HU value of the vertebral cancellous bone was significantly lower than normal, and the amount of bone contained in the vertebral body was significantly reduced. As bone strength decreases, muscle mass and function also decrease, and both bone fragility and the fall risk increase, with osteoporotic fractures as a potential clinical outcome. There are many factors affecting vertebral fractures, as well as the degree of vertebral compression. When a vertebral fragility fracture occurs, the bone mass of the vertebral body is a significant factor affecting the degree of vertebral compression. Our study proves this point using the relationship between the CT HU value and degree of vertebral compression.

At present, many conventional CT studies of osteoporosis fractures at the L1 vertebral level have been performed. Studies [20] have proven that conventional CT-measured CT HU value can be used to identify normal vertebral bone mass and osteoporosis. Emohare et al. [21] reported thoracolumbar compression fractures in elderly patients to have a value of approximately 110 HU, but approximately a quarter of the fractures in the elderly patients in the study were not caused by low-energy trauma. Lee et al. [11] reported the average L1-HU value of vertebral fractures in elderly patients to be approximately 85 HU, which was lower than the average L1-HU value of normal people (approximately 125 HU). However, the use of an enhanced CT contrast agent can increase the L1-HU value by an average of 11 HU compared with that of plain CT [22]. Zou et al. [10] reported that the average L1-HU value of acute vertebral fragility fractures was 66.0 HU by age and sex matching, and the AUC value for predicting vertebral fragility fractures by the vertebral CT value was 0.77 (95% CI 0.70–0.85; *P* < 0.001). The cutoff values of vertebral CT values corresponding to 90% specificity and 90% sensitivity were 60 HU and 100 HU, respectively, in the elderly population; conventional CT measured the CT value of L1 vertebral body trabecular bone, which is closely related to the occurrence of vertebral fracture, and L1 attenuation ≤ 90 HU may represent the best threshold for determining the risk of osteoporotic vertebral fracture [11, 23]. Schreiber et al. [13] used a polyurethane model for dynamic mechanical experiments and proved that the CT HU value is significantly correlated with the compressive strength of bone. Furthermore, our study preliminarily demonstrates that for low-energy fragility fractures in elderly individuals, the lower the CT HU value of cancellous bone is, the more severe the loss of vertebral height; conversely, the higher the CT HU value of cancellous bone is, the less severe the loss of vertebral height.

This study has two limitations. First, the sample size of patients was small because we used strict inclusion criteria for this retrospective investigation. Further longitudinal studies with larger cohorts are needed to verify this relationship. Second, we only studied the effect of the cancellous bone CT HU value on the vertebral body of frangible fractures; however, other studies [24] have shown that compression fractures are associated with paraspinal fractures. Muscles such as the psoas are related to volume reduction. The degree of compression is also associated with other factors (BMI; delay of diagnosis; age et al.), and we did not associate the risk of fracture with low CT HU. Therefore, the factors related to the height loss of vertebral compression fractures are worthy of further study.

## Conclusions

In conclusion, the local bone quality as evaluated by the CT HU value is an important factor affecting the degree of compression in osteoporotic vertebral fractures. This study provides quantitative evidence that a greater compression ratio with thoracolumbar osteoporotic fractures was associated with lower bone density in elderly patients. Further longitudinal studies with larger cohorts are needed to verify this relationship.

## Data Availability

The datasets generated during and/or analyzed during the current study are available in the Guilin Medical University Library's repository [https://mgmt.glmc.edu.cn/tsg/dzzy/zjsjk.htm].

## Abbreviations

DXA: Dual-energy X-ray absorptiometry
BMD: Bone mineral density
ICC: Intraclass correlation coefficient
HU: Hounsfield unit
